# Jot: guiding journal selection with suitability metrics

**DOI:** 10.5195/jmla.2022.1499

**Published:** 2022-07-01

**Authors:** Stephen G. Gaffney, Jeffrey P. Townsend

**Affiliations:** 1 stephen.gaffney@yale.edu, Department of Biostatistics, Yale School of Public Health, Yale University, New Haven, CT.; 2 jeffrey.townsend@yale.edu, Elihu Professor of Biostatistics and Professor of Ecology and Evolutionary Biology; Co-Leader, Genomics, Genetics, & Epigenetics Research Program, Program in Computational Biology and Bioinformatics, Department of Biostatistics, Yale School of Public Health, New Haven, CT.

**Keywords:** Journal selection, web application, bibliometrics, visualization, scientific publishing, open access publishing, open source software, data integration

## Abstract

Researchers grapple with a challenging and consequential decision each time they choose a journal for manuscript submission. There are several online tools that attempt to identify appropriate journals for a manuscript, but each of these tools has shortcomings in terms of the journal data they provide and the exploration functionality they offer—and not one of these tools is open source. Jot is a free and open-source web application that matches manuscripts in the fields of biomedicine and life sciences with suitable journals, based on a manuscript's title, abstract, and (optionally) citations. Jot gathers a wealth of data on journal quality, impact, fit, and open access options that can be explored through a dashboard of linked, interactive visualizations.

## INTRODUCTION

Journal articles are the currency of academic research. The reputation and career advancement of an individual researcher is influenced not only by the importance of their published results, but also by factors such as the number of articles they publish, the number of times their articles are cited, and the prestige of the journals that their articles are published in [1,2]. Publication in journals should give a boost to reputation and career, yet poor selection of a venue for review and publication can detract from the impact of research and call into question its validity, effectively doing the opposite. Predatory journals now number in the thousands [3] and have infiltrated major citation databases [4]; inclusion in a researcher's publication record can become a red flag in a grant application or a review, promotion, or tenure meeting. The decision on which journal to submit to therefore carries high stakes.

Journal selection is not only consequential but also a challengingly complex decision. Journal selection has implications for readership, discoverability, accessibility, preservation, publication costs, time to publication, availability of download statistics, and rights accorded to reader and author [5]. This wide array of journal characteristics presents numerous trade-offs that must be considered in the context of an author's values, the competing priorities of multiple authors, the stipulations of supporting institutions and funding bodies, the urgency of publication, and the particularities of the manuscript in question, which inform journal fit and chance of acceptance. Journal selection is thus a difficult balancing act that precludes a one-size-fits-all ranking system.

To support researchers, tools have been developed that match manuscripts with journals by manuscript title, abstract, and (in some cases) citations [6–8]. These tools vary in matching strategy, in the journal information they provide (such as publication speed, processing charges, and impact metrics), and in which journals are included (e.g., well-marketed tools developed by Elsevier, Springer, Wiley, and IEEE evaluate only the journals that they publish). We have developed a comprehensive tool, Jot, that enters this arena as the sole open-source option, further distinguished by its use of interactive visualizations to explore the many facets of journal metadata, and by a composite metric of journal fit and impact conveying a rough idea of the 'prospect' of a potential submission.

## IMPLEMENTATION

Jot builds upon the API of Jane (Journal/Author Name Estimator) [9]. For a text query, such as title and abstract, Jane uses the Lucene search engine to identify the 50 most-similar recent articles from a filtered set of records drawn from journals indexed within MEDLINE and articles deposited into PubMed Central. This dependence upon MEDLINE and PubMed Central makes Jane—and by extension Jot—most suited to searching the space of biomedical and life sciences journals. Jot runs two queries against the Jane API: the manuscript title and manuscript abstract, gathering the matched articles and similarity scores, before optionally combining them with manuscript citations and a wealth of journal metadata derived from the National Library of Medicine (NLM) Catalog, the Directory of Open Access Journals (DOAJ), Sherpa Romeo, and impact metric databases.

## FEATURES

### Linked plots

Jot uses the Bokeh library [10] to generate pannable, zoomable, interactive visualizations enhanced by hover tooltips. Journals selected in one view become selected in all other views via linked brushing, facilitating multidimensional data exploration. Widgets enable additional personalized functionality such as setting a preferred impact metric and sorting priority.

### Prospect View

**Figure 1:**
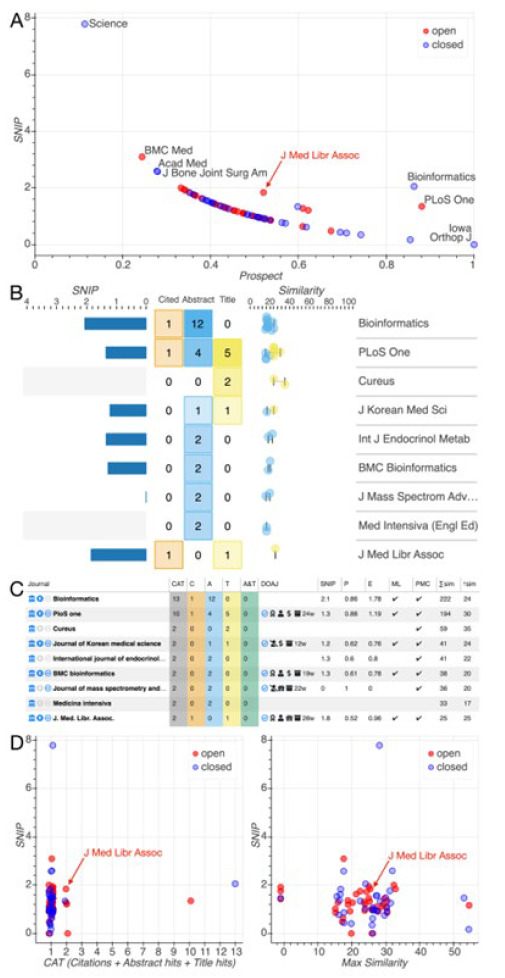
The Jot dashboard contains linked, interactive visualizations to explore characteristics and suitability metrics of potential target journals. The four primary views are (A) Prospect, (B) Articles, (C) Table, and (D) Fit. Shown here are the results for this Jot manuscript (limited to 9 journals in panels B and C), using SNIP as impact metric, and an impact weight of 1.

With the 'prospect' metric:



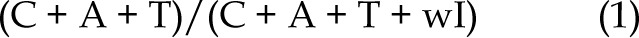



we provide a rough, relative metric of the elusive 'probability of acceptance by a journal, capturing the ratio of 'things in favor of a manuscript' to 'all considerations for or against a manuscript'. In favor of a manuscript are the number of times the manuscript cites the journal (C) and the number of similar article matches for the journal from abstract and title queries (A and T, respectively). The impact (I) of the journal counts against the prospect with a scalar weighting factor (w). Laid out in a scatter plot of prospect vs impact, the Prospect view indicates journals that provide a good fit for submission (Figure 1A). Users can immediately identify the 'dominant' journals (annotated with titles) that provide the highest impact for a given level of prospect.

### Articles View

Similar articles are displayed on a similarity-score axis and colored according to the associated query (abstract or title). C, A, and T are tabulated in a central grid, accompanied by a histogram of impact values (Figure 1B).

### Table View

An interactive table (Figure 1C) provides journal match statistics, impact metrics, and key journal quality indicators: MEDLINE indexing, PubMed Central archival, and inclusion in the Directory of Online Access Journals (DOAJ). DOAJ journals are annotated with copyright ownership, maximum article processing charge, average time to publication, preservation databases, and, when applicable, the DOAJ Seal.

### Fit View

Scatter plots quantify impact versus three metrics of journal relevance, or 'fit': the sum of C, A, and T; the sum of article similarity scores; and the maximum similarity score (Figure 1D).

### OA Pathways

Journals that are not present in DOAJ but that do offer open-access pathways can be identified by following the Sherpa Romeo links in the Table view.

### External Links

Users can navigate to the journal listing in the NLM Catalog or article page in PubMed Central by clicking on the corresponding scatter plot marker.

### Saved Data

Results from the user's most recent search are stored within the browser session, allowing them to be explored again on subsequent visits to Jot. User preferences for impact metric and weight are also stored and used automatically with future searches.

### Offline Browsing or Sharing

A download button on the Results page saves the full Jot dashboard to a single HTML file (with embedded JavaScript).

### Python Package / CLI

Anyone can run—and tinker with—their own Jot server using our open-source Python package journal_targeter (GPLv3 license), available from the Python Package Index (PyPI) and GitHub [11]. The CLI can also update source data and generate HTML results files from command-line inputs.

## USE-CASE

For this very article, we used Jot to identify the Journal of the Medical Library Association (JMLA) as a desirable target journal, and one with which, prior to this work, we were not familiar. We selected a journal-citation metric, Source Normalized Impact per Paper (SNIP), that enables normalized comparisons between journals from different disciplines. JMLA stood out on our plot of prospect (Figure 1A). Indeed, the Articles and Table views (Figure 1B–C) show that JMLA is one of only nine journals with a CAT score (C + A + T) greater than 1. We see in the Table view (Figure 1C) that five of these nine journals share three indicators of quality: they are indexed by MEDLINE, they have a full complement of impact metrics, and they have their open-access policies listed in Sherpa Romeo. Clicking on the ‘E' header to sort by the expected impact (prospect times impact), JMLA is in the top three.

Selecting the five-journal subset identified by the quality indicators and switching back to the Prospect view, we see that they separate from the pack, lifted off the curve of lower-relevance journals (C + A + T = 1; Figure 1A). Continuing to use SNIP as the displayed impact metric, we see that JMLA has above-median impact (SNIP = 1.8) and the highest impact among the fully open-access members of the group (shown with red markers). JMLA is dominated only by the closed-access journal Bioinformatics, with its slightly higher SNIP of 2.1.

Further examination of the articles listed in the Articles view reveals that Bioinformatics articles are matched on the basis of terms relating to open-source software, whereas the title-matched JMLA article is more relevant, as it is on the topic of journal metrics. Valuing open access, we are further encouraged by JMLA's impeccable open-access credentials: the DOAJ column of the table view (Figure 1C) shows that it has been awarded the DOAJ seal, that authors retain unrestricted copyright (corroborated by the linked Sherpa Romeo page), there are no article-processing charges, and articles are preserved in PMC. Undaunted by the expected 26-week submission-to-publication time, we settled on submission to JMLA after confirming compatibility with its aims and scope as stated on the journal web site (linked from the DOAJ page). JMLA's combination of focus and readership seemed an ideal match.

## DISCUSSION

Jot provides a personalized, multi-dimensional analysis that can be navigated through a series of linked, interactive plots and tables, enabling an author to sort and study journals by the potential impact it might have in each journal (based on a diversity of metrics), by the prospect of its acceptance, and according to the publishing attributes most important to them.

Jot reports every journal for which Jane returns a result based on title and abstract queries. Accordingly, Jot inherits aspects of both the journal curation and the textual similarity algorithm that are implemented by Jane. With regard to journal curation, Jane provides a warning that predatory journals can appear among its results, and this warning applies for Jot as well. Following Jane's example, Jot provides indicators of journal quality such as MEDLINE indexing, PMC archival, DOAJ indexing, as well as impact metrics from curated sources such as Scopus and JCR. With regard to the textual similarity algorithm, Jane's implementation assigns importance to words based on frequencies of occurrence in the query text compared to specific journal articles and across the PubMed dataset as a whole. Such word- frequency matching can yield results that would not correspond with human judgment—especially when low-frequency words are inappropriately matched to homographs (words that share spelling but differ in meaning). In our analysis of this manuscript, for example, two out of the three top similarity scores (54.55 and 53.07) stemmed from homographic matches on the word “JOT”—used as an abbreviation for The Journal of Orthopaedic Trauma. Consequently, users should be attentive to the possibility of spurious matches and should examine the article matches that Jot provides to verify the relevance of any suggested journal.

We hope that by sharing Jot with the wider research community—without cost and with the freedom to modify, tailor, and improve it—authors can benefit from a more comprehensive and informed view of their publishing options. Jot has proven useful for our laboratory and has been enthusiastically adopted by our colleagues. One group expressing special excitement about the application during development was health-sciences librarians (Bronars 2013). The librarians we consulted while developing Jot were a wealth of knowledge about the landscape of open access, the consequences of metrics literacy, indicators of discoverability, and database curation and selectivity, and they were highly attuned to the value of tying together multiple sources of journal information. Health-science librarians frequently work with students and early-career researchers seeking guidance on where to publish. Jot could assist this guidance and could also prove useful for librarians seeking to publish their own research. Jot has helped us by directing us to publication of this commentary in the Journal of the Medical Library Association, presenting a high potential to reach a target audience of health science librarians who regularly encounter decisions of journal choice. The potential for a more comprehensive and informed view arising from widespread use of Jot may in turn facilitate more active competition among journals, and therefore desirable evolution of the ever-developing enterprise of scientific publishing and research communication.

## Data Availability

Jot can be used through its website [12], or run as standalone software on Mac, Windows or Linux. Jot is a Flask web application that is controlled through a command line interface. The Python package (journal_targeter) can be installed using pip or pipx [13]. Full source code and documentation is available on GitHub [11] with a GPLv3 license.
